# Mind the weather: a report on inter-annual variations in entomological data within a rural community under insecticide-treated wall lining installation in Kwara State, Nigeria

**DOI:** 10.1186/s13071-018-3078-z

**Published:** 2018-09-04

**Authors:** Abiodun Obembe, Kehinde O. K. Popoola, Adedayo O. Oduola, Samson T. Awolola

**Affiliations:** 1 Department of Biosciences and Biotechnology, Kwara State University Malete, Malete, Nigeria; 2 Department of Zoology, University of Ibadan, Ibadan, Nigeria; 3 Molecular Entomology and Vector Control Research Laboratory, Nigerian Institute of Medical Research, Yaba, Lagos, Nigeria; 4 Department of Zoology, University of Ilorin, Ilorin, Nigeria

**Keywords:** Durable wall lining, Malaria vectors, Rainfall variations

## Abstract

**Background:**

Entomological indices within a specific area vary with climatic factors such as rainfall, temperature and relative humidity. Contributions of such weather parameter fluctuations to the changes in entomological data obtained within a community under implementation of a promising vector control intervention should be taken into account. This study reports on inter-annual changes in entomological indices within two rural communities, one of which was under insecticide-treated durable wall lining (DL) installation.

**Methods:**

Community-wide DL installation was followed by monthly meteorological data and pyrethrum spray mosquito collections for 2 years in intervention and a similar neighbouring community (control). Human blood meal and sporozoite ELISA tests were conducted on female mosquitoes collected alongside PCR identification of subsamples. Mosquitoes collected at the intervention site were tested in cone susceptibility assays against subsamples of installed DL materials collected on a 6-monthly basis for 2 years. Deltamethrin susceptibility of *Anopheles* mosquitoes from the intervention site was determined before and after DL installation. Entomological indices in the first and second years were compared within each site.

**Results:**

Rainfall in the study area increased significantly (*t* = -3.45, *df* = 11, *P* = 0.005) from first to second year. Correlation between rainfall and *Anopheles* densities in both sites were significant (*r* = 0.681, *P* < 0.001). Mosquitoes collected at the intervention site were susceptible (100%) to deltamethrin at baseline but resistant (92%) in the second year. However, subsamples of installed DL materials remained effective (100% mortality) against *Anopheles* mosquitoes from the intervention site throughout the 6-monthly cone assay exposures. Monthly pyrethrum spray collections showed significant increase in *Anopheles* densities from first to second year in the control (6.36 ± 1.61 *vs* 7.83 ± 2.39; *t* = -3.47, *df* = 11, *P* = 0.005), but not in the intervention (2.83 ± 1.86 *vs* 4.23 ± 3.31; *t* = -2.03, *df* = 11, *P* = 0.067) community. However, mean annual mosquito man-biting rates increased significantly in both intervention (0.88 ± 0.18 *vs* 1.06 ± 0.38; *F*_(1, 10)_ = 9.50, *P* = 0.012) and control (1.45 ± 0.31 *vs* 1.61 ± 0.34; *F*_(1, 10)_ = 10.18, *p* = 0.010) sites along with increase (≥ 1.6 times) in sporozoite rates within intervention (0–2.13%) and control (2.56–4.04%) communities.

**Conclusions:**

The slight increase in vector density, induced by significant increase in rainfall, led to increased sporozoite infection and significantly increased man-biting rates within the intervention site. These reveal the need for incorporation of integrated vector management strategies to complement DL installation especially in regions with high rainfall and mosquito density. Promising vector control tools such as DL should be evaluated on a long-term basis to reveal the possible effect of weather parameters on control performance and also allow for holistic recommendations.

**Electronic supplementary material:**

The online version of this article (10.1186/s13071-018-3078-z) contains supplementary material, which is available to authorized users.

## Background

The decline/upsurge of mosquito indices within an area may not be exclusively due to the implementation of community-based vector control tools. Variations in climatic factors such as temperature and rainfall could contribute to changes in entomological indices [1, 2]. However, climate induced changes in entomological data occur between seasons and over years [3, 4] and may not be evident in the results of short term or single year surveys. Consequently, there is the need to conduct long-term entomological surveys in any area under vector control intervention. Outcomes of such surveys will help to profile the contributions of weather fluctuations to entomological changes obtained after vector control. Performance of a promising vector control tool under different seasons and years will also assist in making robust recommendations for future deployments of the tool. This is particularly important now that the need for improved vector control tools is currently being emphasized to sustain the recent decline in the global malaria burden.

Insecticide-treated durable wall lining (DL) is one of such tools still under discussion within the research community. It is expected to outlast indoor residual spray (IRS) in the same way long-lasting insecticidal net (LLIN) circumvents the need for conventional insecticide-treated net (ITN) retreatment [5, 6]. The longest community-based assessments of pyrethroid DL focused on product acceptability and bioefficacy with results of little to no decline in DL bioefficacy over a period of 12–15 months [5, 6]. Moreover, none of the previous DL studies [7–9] considering mosquito vector abundance and infectivity rates were conducted for more than a year to account for the contribution of weather fluctuations to the changes in entomological data obtained within the community under intervention. This study gives an account of variations in endophilic *Anopheles* mosquito vector abundance and infection rates in relation to rainfall within two rural communities, one of which was under village-scale DL installation in Kwara State, Nigeria.

## Methods

### Study area and design

The study was conducted in Kwara State, North Central Nigeria. The State has been classified as derived Savannah, being the transitional zone between the rain forest and arid Sudan Savannah region of Nigeria [10]. The land area covers about 32,500 km^2^ with a population of over two million people [11] spread across sixteen Local Government Areas. Annual rainfall ranges between 1000–1500 mm and maximum average temperature between 30–35 °C [12]. Preliminary visits and assessments were conducted to identify isolated but similar rural communities willing to accept village-wide DL installation. This was based on the report showing the preference of rural communities for the use of DL as against the urban settlements in Nigeria [5]. Akorede (08^o^40.048N, 004^o^31.370E) and Lumoh (08°38.001N, 004°33.740E) which were the most similar communities identified in terms of number of houses (14 and 12), households (50 and 45), human population (167 and 155) and baseline *Anopheles* densities (15.30 and 15.10 per room) were selected as intervention and control communities, respectively. Lumoh community was not a full control treatment due to the unavailability and non-installation of untreated DL in the village. Therefore, entomological parameters considered in this study were not compared between the intervention and control communities. Rather, this study reports changes in entomological indices within the intervention community over seasons and years as influenced by possible loss of DL insecticidal efficacy, insecticide resistance or variations in rainfall. Since neighbouring locations are influenced by similar exposures such as climate and environment due to close proximities [13], entomological indices were also assessed in a similar neighbouring (control) community to confirm whether any climate-induced changes in vector abundance within the intervention village was occurring in any other village in the area at the time.

Both communities are in Moro Local Government Area of Kwara State. Houses in the communities were of the mud type with no ceilings and window nets leaving unrestricted entry points for mosquitoes. The residents of the rural communities were mostly farmers that contribute largely to the production of staple food brought to modern markets within and outside the State. Preliminary surveys conducted in several rural communities during study site selection in the area showed the absence of LLIN in the homes visited. Specifically, all the households in the intervention and control villages were without LLIN. This study was therefore not conducted under a universal or moderate LLIN coverage setting because of the unavailability of such rural communities.

### Insecticide susceptibility tests

Non-blood-fed 2–3 day-old adult female *Anopheles gambiae* (*s.l*.) mosquitoes reared from larval collections at the intervention site were, respectively, exposed before and after DL installation in the second year to test papers containing diagnostic deltamethrin concentrations following standard procedures [14]. Mosquitoes exposed at baseline (100 mosquitoes, 4 replicates) and in the second year (200 mosquitoes, 8 replicates) were transferred into holding tubes, supplied with 10% sugar solution and monitored for mortality 24 h after exposure.

### Durable lining installation and susceptibility of *Anopheles* from the intervention site to DL

The deltamethrin (4.4 g/kg ± 15% a.i.) incorporated DL (100% polyethylene) was provided by Vestergaard Frandsen (Lausanne, Switzerland) and installed in all available sleeping rooms in the intervention community. The DL materials were measured and cut according to dimensions of the walls in each room and installed with the capped nails supplied with the lining. Wall-linings were fixed starting from the highest part of the wall and stretched until it is made straight with the bottom part that was folded inwards in cases where the walls were low. The installation was conducted in September 2012 after baseline mosquito collections. Only three unavailable households were not covered with DL out of the 50 households in the village. The inhabitants of the uncovered households had moved out of the village with the houses locked before the commencement of the study. Each subsample (50 × 50 cm) of already installed DL materials collected from ten randomly selected rooms were tested against 45 non-blood-fed 2–3 day-old female *An. gambiae* (*s.l.*) (five samples/cone, 9 replicates/DL), reared from larval collections at the intervention site, to ascertain continued insecticidal efficacy immediately after installation and at 6 months intervals following standard procedures [15]. The bare wall spaces were replaced with new DL materials. Mosquitoes used for the insecticide susceptibility tests and the continued DL efficacy monitoring were collected from the same larval breeding site at the intervention village. Tracking and visual inspection of DL were conducted during monthly evaluations to identify physical damages or outright removal in the intervention village. Apart from dusts gathering on linings, all the wall-installed DL were intact with no evidence of tear all through the 2 years of the study.

### Mosquito collection and identification

Monthly *Anopheles* mosquito collections were conducted in ten houses in each of the intervention and control communities from October 2012 to September 2014 using Pyrethrum Spray Catch method [16]. One room was chosen per house and the same rooms were sampled throughout the study. The ten rooms were selected based on the willingness of the occupants to allow the use of the rooms throughout the period of the study. The number of persons sleeping in the rooms the night preceding the mosquito collections was also noted for indirect man-biting rates estimations [16–18]. Mosquito samples were preserved on silica for further processing at The Molecular Entomology and Vector Control Research Laboratory of the Nigerian Institute of Medical Research. All samples were identified using morphological keys [19] with subsamples subjected to species-specific PCR [20] and PCR-RFLP [21] assays for sibling species identification.

### Detection of human blood and *P. falciparum* sporozoite infection in mosquitoes

*Plasmodium falciparum* circumsporozoite ELISA [22] were done on heads-thoraces of all the female *Anopheles gambiae* (*s.l.*) mosquito samples collected. Blood-fed mosquitoes were used for human blood meal ELISA [23]. Monoclonal antibodies and positive controls for the sporozoite ELISA were supplied by Centers for Disease Control and Prevention (Atlanta, USA). Human serum and monoclonal antibodies for blood meal ELISA were obtained from Rockland immunochemicals (Gilbertsville, USA) and Kikergaard and Perry Laboratories (Gaithersburg, USA), respectively.

### Collection of rainfall data

Data on monthly rainfall in the state during the period of the mosquito collection were obtained from the Nigerian Meteorological Agency station at the Ilorin International Airport.

### Data analyses

*Anopheles* densities were taken as the numbers of female *Anopheles gambiae* (*s.l.*) mosquitoes collected from the ten rooms surveyed in each site per unit time. Man-biting rates (MBR) were determined as the number of blood-fed and half-gravid female *Anopheles* samples collected divided by the number of persons sleeping in the rooms the night preceding the collection multiplied by human blood index [16–18]. Human blood index (HBI) was determined as proportion of blood-fed and half-gravid samples with human blood. The number of people in the rooms was taken as a reflection of human availability for mosquito bites because the persons in both villages were not sleeping under a bednet. Data obtained (*Anopheles* densities, man-biting rates and rainfall values) were transformed [√n+0.5] to accommodate zero values and attain normal distribution [24]. Sporozoite infection rates of the indoor resting mosquitoes collected were taken as percentage of female *Anopheles* found with *Plasmodium falciparum* circumsporozoite protein. Mean densities of female *Anopheles gambiae* (*s.l.*) mosquitoes collected in the first and second years were compared within each site using Student’s t*-*test (SPSS 16 software) to determine the significance (*P* < 0.05) of differences. First and second year mean man-biting rates were also compared within each site using logistic regression. Monthly *Anopheles* densities for both sites were pooled and correlated (Pearson’s correlation) with monthly rainfall values obtained for the state during the study period. Rainfall measurements were not taken in the study communities but the Average State Rainfall is believed to reflect the general trend of rainfall in the study villages.

## Results

### Rainfall and *Anopheles* densities

**Fig. 1:**
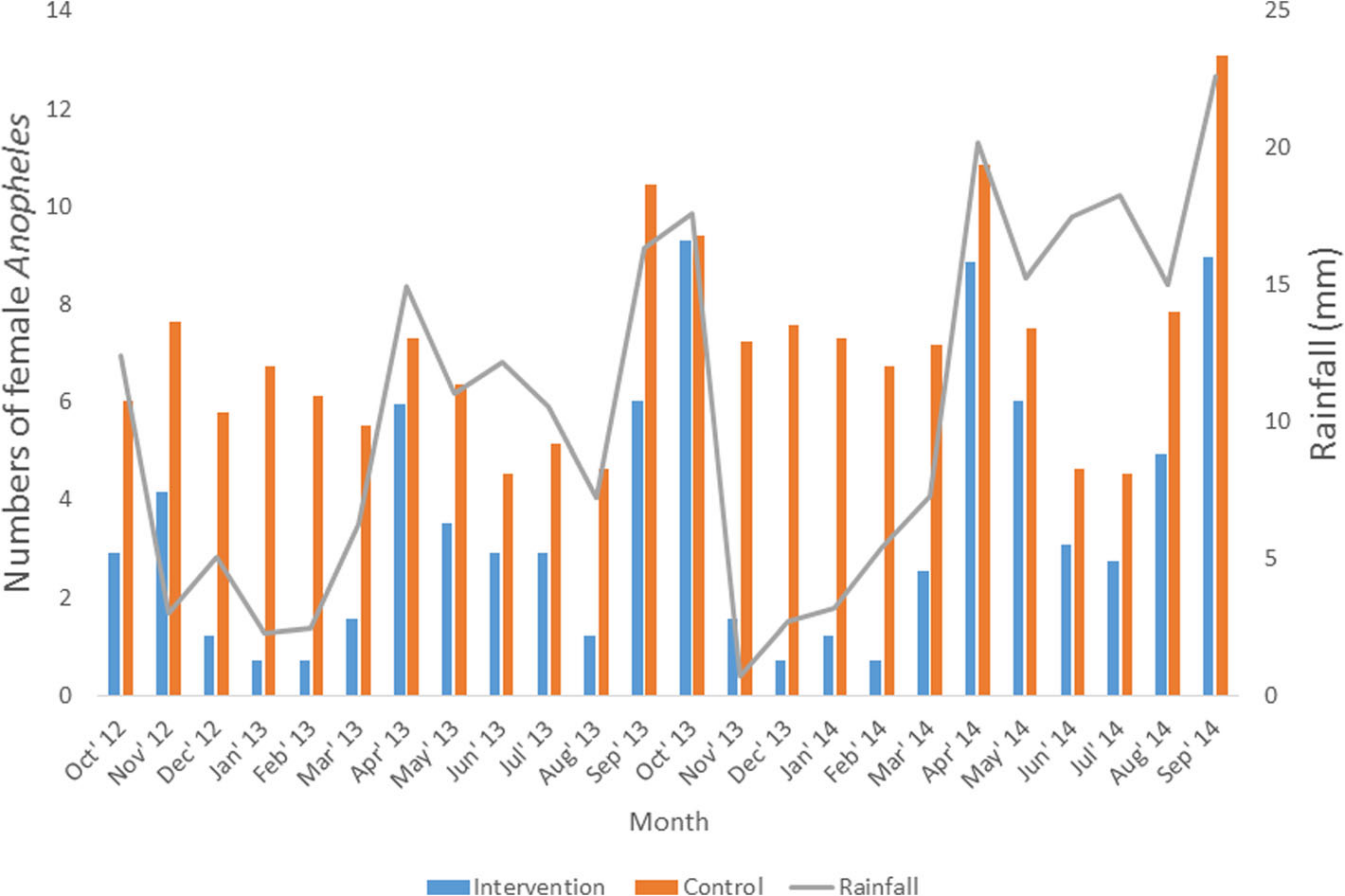
Relationship between rainfall and numbers of female *Anopheles* in the study communities

**Fig. 2:**
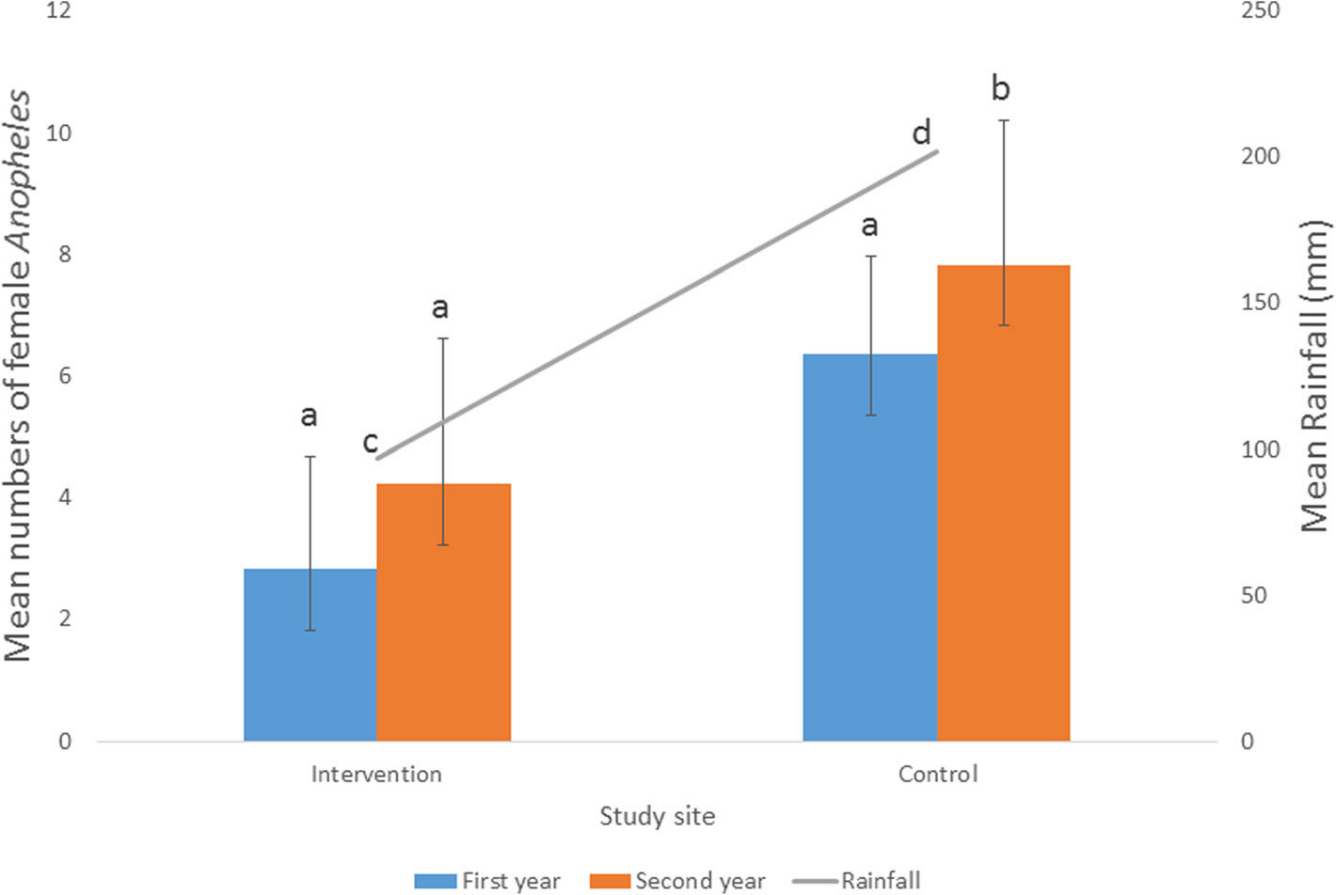
Mean ± standard error (SE) annual rainfall and numbers of female *Anopheles* mosquitoes collected during first and second years in the study communities. Means with the different letters within the same site are significantly different (Student’s t-test, *P* < 0.05)

Wet season (*t* = -10.29, *df* = 5, *P* < 0.001) and annual (*t* = -3.45, *df* = 11, *P* = 0.005) mean rainfall were significantly higher in the second year compared to first year (Table 1). Highest numbers of mosquitoes occurred during highest rainfall (April, September and October) period except for June and July (Fig. 1). Significant correlation (*r* = 0.681, *P* < 0.001) was found between rainfall and *Anopheles* densities in the study area. (Table 2). Mean densities of *Anopheles* collected were significantly higher in the second year than the first year in the control (6.36 ± 1.61 *vs* 7.83 ± 2.39; *t* = -3.47, *df* = 11, *P* = 0.005) but not in the intervention site (2.83 ± 1.86 *vs* 4.23 ± 3.31; *t* = -2.03, *df* = 11, *P* = 0.067) (Fig. 2).

**Table 1:** Rainfall recorded in the area during the study period. Square-root transformation formula X^1^= √X+0.5, where X^1^ is the transformed value and X is the actual value

Period	First year		Second year	
	Actual values	Transformed values	Actual values	Transformed values
Dry season				
October	153.7	12.42	308.8	17.59
November	8.7	3.03	0.0	0.71
December	25.2	5.07	6.8	2.70
January	4.8	2.30	9.7	3.19
February	5.5	2.45	28.6	5.39
March	38.4	6.24	52.2	7.26
Mean ± SD		5.25 ± 3.84		6.14 ± 6.05
Wet season				
April	223.4	14.96	408.8	20.23
May	121.7	11.05	232.3	15.26
June	148.4	12.20	305.2	17.48
July	110.6	10.54	332.3	18.24
August	51.4	7.20	225.3	15.03
September	266.5	16.34	511.7	22.63
Mean ± SD		12.05 ± 3.28		18.15 ± 2.93
Annual mean ± SD		8.65 ± 4.92		12.14 ± 7.74

The difference between first and second year mean rainfall values was significant in the wet (*P* < 0.001) but not in the dry (*P* = 0.494) season. Annual mean rainfall value was significantly (*P* = 0.005) higher in the second year compared to the first year

**Table 2:** Correlation (*r* = 0.681, *P* < 0.001) between rainfall and female *Anopheles* density in the study area. Square root transformation formula X^1^= √X+0.5, where X^1^ is the transformed value and X is the actual value

Month	*Anopheles* density					
	Actual values in intervention site	Transformed values in intervention site	Actual values in control site	Transformed values in control site	Total *Anopheles* densities in both sites	Rainfall (mm)
First year						
October 2012	8	2.92	36	6.04	8.96	12.42
November 2012	17	4.18	58	7.65	11.83	3.03
December 2012	1	1.22	33	5.79	7.01	5.07
January 2013	0	0.71	45	6.75	7.46	2.30
February 2013	0	0.71	37	6.12	6.83	2.45
March 2013	2	1.58	30	5.52	7.10	6.24
April 2013	35	5.96	53	7.31	13.27	14.96
May 2013	12	3.54	40	6.36	9.90	11.05
June 2013	8	2.92	20	4.53	7.45	12.20
July 2013	8	2.92	26	5.15	8.07	10.54
August 2013	1	1.22	21	4.64	5.86	7.20
September 2013	36	6.04	109	10.46	16.50	16.34
Total	128	33.92	508	76.32	110.24	103.80
Second year						
October 2013	86	9.30	88	9.41	18.71	17.59
November 2013	2	1.58	52	7.25	8.83	0.71
December 2013	0	0.71	57	7.58	8.29	2.70
January 2014	1	1.22	53	7.31	8.53	3.19
February 2014	0	0.71	45	6.75	7.46	5.39
March 2014	6	2.55	51	7.18	9.73	7.26
April 2014	78	8.86	117	10.84	19.70	20.23
May 2014	36	6.04	56	7.52	13.56	15.26
June 2014	9	3.08	21	4.64	7.72	17.48
July 2014	7	2.74	20	4.53	7.27	18.24
August 2014	24	4.95	61	7.84	12.79	15.03
September 2014	80	8.97	171	13.09	22.06	22.63
Total	329	50.71	792	93.94	144.65	145.71

### Man-biting and sporozoite infection rates of indoor resting female *Anopheles*

Monthly man-biting rates (Additional file 1: Table S1) derived from *Anopheles* mosquito human blood index (Additional file 2: Table S2) were mostly higher in the first compared to second year. Mean man-biting rates of *Anopheles* mosquitoes increased significantly from first to second year in both intervention sites (0.88 ± 0.18 *vs* 1.06 ± 0.38; *F*_(1, 10)_ = 9.50, *P* = 0.012) and control (1.45 ± 0.31 *vs* 1.61 ± 0.34; *F*_(1, 10)_ = 10.18, *P* = 0.010) (Table 3). Sporozoite rates increased from first to second year by at least 1.6 times in intervention (0–2.13%) and control (2.56–4.04%) communities (Table 4). Only one *An. coluzzii* sample was found with *P. falciparum* sporozoites in the control community while all the infected mosquitoes recorded in the intervention village were identified as *An. gambiae*.

**Table 3:** Man-biting rates of female *Anopheles* mosquitoes in the study sites

Period	Intervention site				Control site			
	Actual values		Transformed values		Actual values		Transformed values	
	Year I	Year II	Year I	Year II	Year I	Year II	Year I	Year II
October	0.21	1.99	0.84	1.58	1.38	2.96	1.37	1.86
November	0.53	0.10	1.01	0.77	2.33	1.44	1.68	1.39
December	0.03	0.00	0.73	0.71	1.50	2.01	1.41	1.58
January	0.00	0.05	0.71	0.74	1.31	1.70	1.35	1.48
February	0.00	0.00	0.71	0.71	1.34	1.49	1.36	1.41
March	0.08	0.25	0.76	0.87	1.39	1.86	1.37	1.54
April	0.71	1.57	1.10	1.44	1.88	3.30	1.54	1.95
May	0.37	0.95	0.93	1.20	1.86	2.13	1.54	1.62
June	0.25	0.28	0.87	0.88	0.81	0.78	1.14	1.13
July	0.28	0.24	0.88	0.86	0.94	0.89	1.20	1.18
August	0.04	0.84	0.73	1.16	0.86	2.73	1.17	1.79
September	1.19	2.77	1.30	1.81	4.76	4.97	2.29	2.34
Mean ± SD			0.88 ± 0.18	1.06 ± 0.38			1.45 ± 0.31	1.61 ± 0.34

Mean man-biting rates were significantly higher in the second year compared to the first year in both intervention (*P* = 0.012) and control (*P* = 0.010) sites

**Table 4:** Sporozoite infection rates of endophilic *Anopheles* in the study sites

Study site	Year	Total no. of female *Anopheles*	No. of *Anopheles* positive for CSP			Sporozoite rates of mosquitoes (%)
			*An. gambiae*	*An. coluzzii*	Total	
Intervention	First	128	0	0	0	0
	Second	329	7	0	7	2.13
Control	First	508	13	0	13	2.56
	Second	792	31	1	32	4.04

### Insecticide resistance and mosquito susceptibility to installed DL

*Anopheles* mosquito population from the intervention site were susceptible (100%) at baseline but resistant (92% mortality, see Additional file 3: Table S3) in the second year. However, 100% knockdown and mortality rates were recorded in the WHO cone susceptibility assay involving exposure of *Anopheles* population from the intervention site to subsamples of the installed DL all through the two-year period of the study.

### *Anopheles* species composition

*Anopheles gambiae* was the predominant mosquito species in the intervention (85% and 90.8%) and control (74% and 87.9%) sites during the first and second years, respectively (Table 5). Only four *An. arabiensis* were identified in the two years of mosquito sampling in the control community.

**Table 5:** Species composition of female *Anopheles* mosquito samples from the study sites

Period	Intervention, *n* (%)				Control, *n* (%)			
	No. assayed	*An. arabiensis*	*An. coluzzii*	*An. gambiae*	No. assayed	*An. arabiensis*	*An. coluzzii*	*An. gambiae*
First year	97	0 (0)	15 (15)	82 (85)	311	2 (0.6)	79 (25.4)	230 (74)
Second year	130	0 (0)	12 (9.2)	118 (90.8)	323	2 (0.6)	37(11.5)	284 (87.9)

## Discussion

This study elucidates dynamics of malaria entomological indices in a community under DL installation in Kwara State, North Central Nigeria. Sustained insecticidal efficacy (100% mortality) of installed DL could be due to reduced contact irritability [25] and higher insecticide dose uptake of the resistant mosquitoes [26] upon exposure to pyrethroids-treated surfaces. The continued efficacy of installed DL against resistant *Anopheles* population suggests that the increase in entomological indices (DL performance) in the second year of this study was not largely as a result of insecticide resistance or loss of DL efficacy. Rather, entomological indices increased with increase in rainfall from the first to the second year as evident also in the control community. This result agrees with the observation of continued efficacy of insecticide-treated materials (LLIN) after the development of resistance in the prevailing mosquitoes in the target community [26, 27]. Yet, it is important to maintain susceptibility of *Anopheles* mosquitoes to pyrethroids, the only class of insecticide currently approved for treating bednets [28]. Therefore, more efforts on development and assessments of non-pyrethroid DL is recommended for resistance management especially as insecticide resistance is currently becoming widespread and gaining potency in the *An. gambiae* mosquitoes [29, 30].

The significant correlation between rainfall and *Anopheles* densities could be linked to the availability of more rain-fed mosquito breeding sites during the rainy season period. Olayemi & Ande [31] had earlier correlated rainfall with mean monthly mosquito abundance in Kwara State. Osse et al. [32] also reported higher human biting rate in second year compared to the first as a result of higher rainfall in some communities under IRS in Benin. However, the impact of DL installation in the intervention community was evident as significant (*P* = 0.005) increase in rainfall did not translate to significant (*P* = 0.067) increase in *Anopheles* densities from first to second year. An exception to the association between rainfall and *Anopheles* densities was found in the mid-rainy season (June-July) of both years when high rainfall and low numbers of *Anopheles* were recorded in both study sites. This is probably due to the high frequency of rainfall which often agitates and washes away the larval breeding sites. This observation has important implications in the event of scarce mosquito control resources expected to be targeted at the peak period of mosquito abundance.

*Anopheles arabiensis* absence and negligible occurrence in the intervention (0/227) and control communities (4/634) is expected since the study area does not fall within the arid regions where this species is known to be abundant [19]. *Anopheles* man-biting rates increased significantly with rainfall despite mosquito density increase that was not significant in the intervention community from first to second year. This could be due to the strong anthropophagic tendencies of the prevailing *An. gambiae* and *An. coluzzii* species in this village, as revealed by the high human blood indices of the mosquito samples. The result therefore shows the need for longitudinal DL evaluation over seasons and years to allow for additional recommendations such as the incorporation of larval management strategies especially in situations of high rainfall and *Anopheles* mosquito densities. This study is limited in that the findings may apply less under the recommended universal LLIN coverage community settings if and when it is sustainably achieved. Expected risks of transmission (EIR) could not be estimated due to pyrethrum spray collection method which collects indoor resting and not host-seeking mosquitoes.

## Conclusions

This study reports on dynamics of malaria entomological indices within a community under deltamethrin durable wall lining installation in Akorede, Kwara State, Nigeria. The result showing no significant increase in *Anopheles* density within the intervention village, despite significant increase in rainfall and mosquito density within the neighbouring control village, suggests DL mortality effects as evidenced by 100% wild mosquito cone bioassays mortality results. However, the insignificant increase in *Anopheles* density was accompanied by increased sporozoite infection (0 to 2%) and significantly increased man-biting rates within the intervention site. This shows the sensitivity of these indices to slight changes in vector density and therefore calls for the incorporation of integrated vector management strategies to complement DL installation especially in regions with abundant rainfall and high mosquito density. The use of non-pyrethroid DL will be worthwhile to restore and preserve vector susceptibility to pyrethroid insecticide currently being used for treatments of long-lasting insecticidal nets.

## Additional files

Additional file 1: **Table S1.** Actual female *Anopheles* man-biting rate calculations in the intervention and control communities. (DOCX 18 kb)

Additional file 2: **Table S2.** Human blood indices of female *Anopheles* mosquitoes in the intervention and control communities. (DOCX 17 kb)

Additional file 3: **Table S3.** Insecticide susceptibility results. Number of dead/alive mosquitoes at the end of holding period (24 h). (DOCX 13 kb)

## Abbreviations

ELISA: enzyme-linked immunosorbent assay
PCR: polymerase chain reaction
DL: insecticide-treated durable wall lining
ITPS: insecticide-treated plastic sheeting
HDPE: high density polyethylene
IRS: indoor residual spray
LLIN: long-lasting insecticidal net
EIR: entomological inoculation rate
NPC: National Population Commission
NIMET: Nigerian Meteorological Agency
RFLP: restriction fragment length polymorphism
SPSS: Statistical Package for the Social Sciences
NIMR: Nigerian Institute of Medical Research
NMEP: National Malaria Elimination Program
WHO: World Health Organization
CSP: circumsporozoite protein
