# Supplementary material for: Mind the weather: a report on inter-annual variations in entomological data within a rural community under insecticide-treated wall lining installation in Kwara State, Nigeria
Source: Parasit Vectors. 2018 Sep 4;11:497. doi: 10.1186/s13071-018-3078-z (PMC6123909; doi:10.1186/s13071-018-3078-z)
Supplement: Supplementary file 1 — Table S1. Actual female Anopheles man-biting rate calculations in the intervention and control communities. (DOCX 18 kb) [file 13071_2018_3078_MOESM1_ESM.docx]

**Additional file 1: Table S1.** Actual female *Anopheles* man-biting rate calculations in the intervention and control communities**.**

|  | **First year** | | | | | | | | **Second year** | | | | | | | |
| --- | --- | --- | --- | --- | --- | --- | --- | --- | --- | --- | --- | --- | --- | --- | --- | --- |
|  | **Intervention** | | |  | **Control** | | |  | **Intervention** | | |  | **Control** | | |  |
| Month | No of fed *An* | No of  sleepers | HBI | MBR | No of  fed *An* | No of  sleepers | HBI | MBR | No of  fed *An* | No of  Sleepers | HBI | MBR | No of  fed *An* | No of  Sleepers | HBI | MBR |
| Oct | 7 | 33 | 1.00 | 0.21 | 34 | 21 | 0.85 | 1.38 | 83 | 33 | 0.79 | 1.99 | 88 | 22 | 0.74 | 2.96 |
| Nov | 16 | 30 | 1.00 | 0.53 | 55 | 21 | 0.89 | 2.33 | 2 | 20 | 1.00 | 0.10 | 52 | 25 | 0.69 | 1.44 |
| Dec | 1 | 30 | 1.00 | 0.03 | 33 | 20 | 0.91 | 1.50 | 0 | 29 | 0.00 | 0.00 | 57 | 23 | 0.81 | 2.01 |
| Jan | 0 | 24 | 0.00 | 0.00 | 45 | 23 | 0.67 | 1.31 | 1 | 22 | 1.00 | 0.05 | 53 | 23 | 0.74 | 1.70 |
| Feb | 0 | 26 | 0.00 | 0.00 | 37 | 21 | 0.76 | 1.34 | 0 | 23 | 0.00 | 0.00 | 45 | 23 | 0.76 | 1.49 |
| Mar | 2 | 25 | 1.00 | 0.08 | 30 | 18 | 0.83 | 1.39 | 6 | 24 | 1.00 | 0.25 | 51 | 23 | 0.84 | 1.86 |
| Apr | 35 | 28 | 0.57 | 0.71 | 53 | 24 | 0.85 | 1.88 | 74 | 32 | 0.68 | 1.57 | 114 | 29 | 0.84 | 3.30 |
| May | 11 | 22 | 0.73 | 0.37 | 40 | 19 | 0.88 | 1.86 | 36 | 26 | 0.69 | 0.95 | 54 | 20 | 0.79 | 2.13 |
| Jun | 6 | 24 | 1.00 | 0.25 | 20 | 16 | 0.65 | 0.81 | 7 | 25 | 1.00 | 0.28 | 20 | 18 | 0.70 | 0.78 |
| Jul | 7 | 25 | 1.00 | 0.28 | 26 | 18 | 0.65 | 0.94 | 6 | 25 | 1.00 | 0.24 | 20 | 18 | 0.80 | 0.89 |
| Aug | 1 | 26 | 1.00 | 0.04 | 21 | 22 | 0.90 | 0.86 | 24 | 25 | 0.88 | 0.84 | 61 | 19 | 0.85 | 2.73 |
| Sep | 33 | 22 | 0.79 | 1.19 | 107 | 20 | 0.89 | 4.76 | 80 | 26 | 0.90 | 2.77 | 171 | 31 | 0.90 | 4.97 |

Man-biting rates (MBR) = no of fed *An*. samples/no of sleepers multiplied by the human blood index (HBI). MBR with the different letters are significantly different (student *t*-test, *P*<0.05)
