# Supplementary material for: Mind the weather: a report on inter-annual variations in entomological data within a rural community under insecticide-treated wall lining installation in Kwara State, Nigeria
Source: Parasit Vectors. 2018 Sep 4;11:497. doi: 10.1186/s13071-018-3078-z (PMC6123909; doi:10.1186/s13071-018-3078-z)
Supplement: Supplementary file 2 — Table S2. Human blood indices of female Anopheles mosquitoes in the intervention and control communities. (DOCX 17 kb) [file 13071_2018_3078_MOESM2_ESM.docx]

**Additional file 2: Table S2.** Human blood indices of female *Anopheles* mosquitoes in the intervention and control communities.

|  | **First year** | | | | | | **Second year** | | | | | |
| --- | --- | --- | --- | --- | --- | --- | --- | --- | --- | --- | --- | --- |
|  | **Intervention** | | | **Control** | | | **Intervention** | | | **Control** | | |
| Month | No. of female *An*  with human blood | Total no. of  female *An*  with blood | **Human blood**  **index** | No. of  female *An*  with human blood | Total no. of  female *An*  with blood | **Human blood**  **index** | No. of  female *An*  with human blood | Total no. of  female *An*  with blood | **Human blood**  **index** | No. of  female *An*  with human blood | Total no. of  female *An*  with blood | **Human blood**  **index** |
| Oct | 7 | 7 | 1.00 | 29 | 34 | 0.85 | 66 | 83 | 0.79 | 65 | 88 | 0.74 |
| Nov | 16 | 16 | 1.00 | 47 | 55 | 0.89 | 2 | 2 | 1.00 | 36 | 52 | 0.69 |
| Dec | 1 | 1 | 1.00 | 30 | 33 | 0.91 | 0 | 0 | 0.00 | 46 | 57 | 0.81 |
| Jan | 0 | 0 | 0.00 | 30 | 45 | 0.67 | 1 | 1 | 1.00 | 39 | 53 | 0.74 |
| Feb | 0 | 0 | 0.00 | 28 | 37 | 0.76 | 0 | 0 | 0.00 | 34 | 45 | 0.76 |
| Mar | 2 | 2 | 1.00 | 25 | 30 | 0.83 | 6 | 6 | 1.00 | 43 | 51 | 0.84 |
| Apr | 20 | 35 | 0.57 | 45 | 53 | 0.85 | 50 | 74 | 0.68 | 96 | 114 | 0.84 |
| May | 8 | 11 | 0.73 | 35 | 40 | 0.88 | 25 | 36 | 0.69 | 43 | 54 | 0.79 |
| Jun | 6 | 6 | 1.00 | 13 | 20 | 0.65 | 7 | 7 | 1.00 | 14 | 20 | 0.70 |
| Jul | 7 | 7 | 1.00 | 17 | 26 | 0.65 | 6 | 6 | 1.00 | 16 | 20 | 0.80 |
| Aug | 1 | 1 | 1.00 | 19 | 21 | 0.90 | 21 | 24 | 0.88 | 52 | 61 | 0.85 |
| Sep | 26 | 33 | 0.79 | 95 | 107 | 0.89 | 72 | 80 | 0.90 | 154 | 171 | 0.90 |

Human blood index=No. of female *Anopheles* with human blood/total no of female *Anopheles* with blood. Human blood index with the same letters are not significantly different (student *t*-test, *P*>0.05).
