# Supplementary material for: Mind the weather: a report on inter-annual variations in entomological data within a rural community under insecticide-treated wall lining installation in Kwara State, Nigeria
Source: Parasit Vectors. 2018 Sep 4;11:497. doi: 10.1186/s13071-018-3078-z (PMC6123909; doi:10.1186/s13071-018-3078-z)
Supplement: Supplementary file 3 — Table S3. Insecticide susceptibility results. Number of dead/alive mosquitoes at the end of holding period (24 h). (DOCX 13 kb) [file 13071_2018_3078_MOESM3_ESM.docx]

Additional file 3: Table S3. Number of dead/alive mosquitoes at the end of holding period (24 hours)

|  | Rep 1 | Rep 2 | Rep 3 | Rep 4 | Rep 5 | Rep 6 | Rep 7 | Rep 8 | Total | Control 1 | Control 2 |
| --- | --- | --- | --- | --- | --- | --- | --- | --- | --- | --- | --- |
| No. dead | 23 | 22 | 23 | 23 | 24 | 23 | 23 | 23 | 184 | 0 | 0 |
| No. alive | 2 | 3 | 2 | 2 | 1 | 2 | 2 | 2 | 16 | 25 | 25 |
| Total | 25 | 25 | 25 | 25 | 25 | 25 | 25 | 25 | 200 | 25 | 25 |

Percentage mortality = Total number of dead mosquitoes / total number of mosquitoes exposed × 100.

Percentage mortality = 184/200 × 100 = 92%.
